# Does ‘mapping’ the evidence usefully inform the research question prioritisation process in systematic reviews?

**DOI:** 10.1186/1745-6215-16-S2-P170

**Published:** 2015-11-16

**Authors:** Caroline Main, Jayne Wilson, Martin English, Pamela Kearns, Bob Phillips, Barry Pizer, Sophie Wilne, Keith Wheatley

**Affiliations:** 1 University of Birmingham, Birmingham, UK; 2 Birmingham Children's Hospital NHS Foundation Trust, Birmingham, UK; 3 Centre for Reviews and Dissemination (CRD), York, UK; 4 Alder Hey Children's NHS Foundation Trust, Liverpool, UK; 5 Nottingham University Hospitals’ NHS Trust, Nottingham, UK

## Background

Defining the research questions in systematic reviews is a very important first stage in the process, providing the framework for subsequent stages. With numerous questions and uncertainty regarding the strength and size of the evidence base for undertaking reviews on the effectiveness of interventions for children's central nervous system (CNS) tumours a ‘map’ of the evidence was produced.

## Methods

Ten electronic databases were searched for published and on-going studies on surgical procedures, radiotherapy (RT), chemotherapy (CT), hormone therapy (HT), immunotherapy, biological therapies and imaging from 1985 to November 2014. No study design filters were applied. Studies were categorised as relevant interventions, or excluded due to the patient population or lack of relevant intervention. All included studies were then categorised by tumour histology type, intervention and study design on the basis of the available abstract.

## Results

A total of 8,448 references were identified; with 1,665 studies included in the ‘map’. Twelve systematic reviews, 30 randomised controlled trials (RCTs) and 200 single arm phase II trials with 8785 participants were identified. The majority of the evidence was limited to case series studies (1,076) or case reports with less than 5 patients (306). Chemotherapy regimens, either alone or in combination with RT were the most frequently assessed interventions. There were very few studies of HT, immunotherapy or biological agents identified.

## Conclusions

‘Mapping’ where evidence is scarce or of a lower quality is a useful tool for prioritising research questions, re-defining initial conceptualisation of questions, highlighting gaps and prioritising review schedules when emerging technologies are being assessed.

